# 

**Published:** 1908-09

**Authors:** 


### CONTENTS.

ORIGINAL COMMUNICATIONS—
Some Aspects of Intestinal Auto-Intoxication with Report of Two Cases Showing Unusual Skin Manifestations. By Lewis M. Gaines, A. B., M. D., Atlanta, Ga. .. .. .. .. .. .. 300
Hydatidform Mole (Myxoma Chorii), with Report of Case. By J. B. Cranner, M. D., Wilmington, N. C. .. .. .. .. .. .. .. .. .. .. .. .. 304
Advantages of Early Operations in Appendicitis—Report of Cases. By Jas. L. Campbell, M. D. .. .. .. .. .. .. .. .. .. .. .. 307
Abscess of the Brain. By. R. C. Buckner, Ashville, N. C. .. .. .. .. .. .. .. .. 312
Report of a Case of Suppurative Leiomyoma of Stomach. Hour Glass Contraction of Same. By Whatley W. attey, Jr., M. D., Augusta, Ga. .. .. .. .. .. .. .. 326
Post-Graduate Study in Europe. With special Reference to Eye, Ear, Nose and Throat. Otis H. Johnson, B. A., M. D., Athens, Ga. .. .. .. .. .. .. .. .. 329
EDITORIALS .. .. .. .. .. .. .. .. .. .. .. .. 338
NEWS AND NOTES .. .. .. .. .. .. .. .. .. .. .. .. 341
FULTON CAOUNTY MEDICAL SOCIETY .. .. .. .. .. .. .. .. .. .. 344
BOOKS RECEIVED .. .. .. .. .. .. .. .. .. .. .. .. 348
BOOK REVIEWS .. .. .. .. .. .. .. .. .. .. .. .. 349

